# Editorial: Prediabetes: new insights on the diagnosis, risk stratification, comorbidites, cardiovascular disease, microvascular complications, and treatment

**DOI:** 10.3389/fendo.2023.1214479

**Published:** 2023-05-11

**Authors:** João Sérgio Neves, Martin Buysschaert, Michael Bergman

**Affiliations:** ^1^ Department of Endocrinology, Diabetes and Metabolism, Centro Hospitalar Universitário de São João, Porto, Portugal; ^2^ Cardiovascular Research and Development Center, Department of Surgery and Physiology, Faculty of Medicine, University of Porto, Porto, Portugal; ^3^ Department of Endocrinology and Diabetology, Université Catholique de Louvain, University Clinic Saint-Luc, Brussels, Belgium; ^4^ Division of Endocrinology, Diabetes and Metabolism, New York University Grossman School of Medicine, New York, NY, United States

**Keywords:** prediabetes, impaired glucose tolerance, impaired fasting glucose, cardiovascular disease, microvascular complications

Prediabetes is a highly prevalent condition affecting more than one-third of adults in the US and more than one-fifth in Europe (1, 2). In addition to the well-known increased risk of progression to diabetes, prediabetes is also associated with an increased risk of cardiovascular complications (3). The focus on mechanisms and interventions to reduce the risk of cardio-renal complications in prediabetes is markedly less than that on preventing diabetes (3). More research is needed to better characterize new markers of microvascular and macrovascular risk in prediabetes and to develop new interventions to minimize this risk. In the Research Topic “*Prediabetes: New Insights on Diagnosis, Risk Stratification, Comorbidities, Cardiovascular Disease, Microvascular Complications, and Treatment*” the authors have made significant contributions to the field of prediabetes (Shu et al., Wang et al., Cloro et al., Wang et al., Zhu et al., Wang et al.) (**Figure 1**).

**Figure 1:**
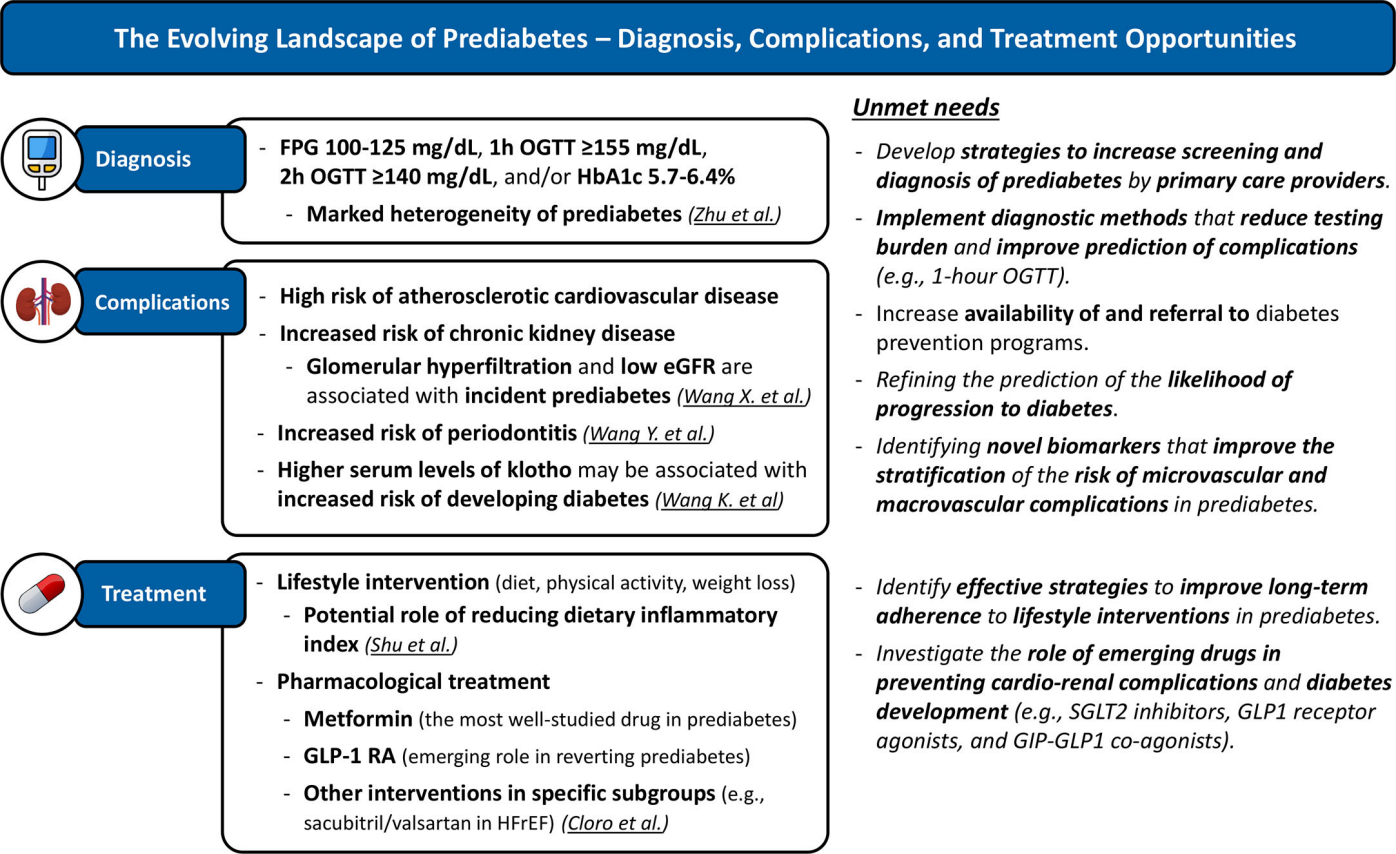
The Evolving Landscape of Prediabetes – Diagnosis, Complications, and Treatment Opportunities. Current knowledge and unmet needs. Underlined text highlights contributions made by articles published in the special topic “Prediabetes: New Insights on Diagnosis, Risk Stratification, Comorbidities, Cardiovascular Disease, Microvascular Complications, and Treatment”. eGFR, estimated glomerular filtration rate; FPG, fasting plasma glucose; GLP-1 RA, GLP-1 receptor agonists; HFrEF, heart failure with reduced ejection fraction; OGTT, oral glucose tolerance test; SGLT2i, SGLT2 inhibitors.

## Prediabetes diagnosis and criteria

Zhu et al. conducted a population-based longitudinal study to identify factors correlated with targeted prevention of prediabetes. This study provides several relevant conclusions. First, consistent with previous reports (Wang et al.), there was a minimal overlap between the different definitions of prediabetes; only about 10% of participants with prediabetes met all three ADA (American Diabetes Association) criteria for prediabetes. Second, adiposity and obesity-related parameters were significantly associated with prediabetes, which further reinforces the central role of obesity for the development of glycemic dysfunction. Lastly, this study revealed interesting patterns in the distribution of prediabetes criteria in the Chinese population studied. Men were more likely to have impaired fasting glucose (IFG), while women were more likely to have impaired glucose tolerance (IGT). Additionally, different anthropometric parameters may be associated with different prediabetes criteria. Overall, these results emphasize the heterogeneity of prediabetes and highlight the need for targeted interventions in the diagnosis and treatment of prediabetes.

## Lifestyle interventions and prediabetes

Shu et al. investigated the role of the dietary inflammatory index in prediabetes and insulin resistance among American adults. Their findings suggest that a pro-inflammatory diet, such as diets rich in processed meats (e.g., bacon) refined carbohydrates (e.g., white bread) or sugar-sweetened beverages, is associated with higher fasting plasma glucose (FPG), fasting serum insulin, and HOMA-IR, and consequently increases the odds of prediabetes and insulin resistance. These results suggest that dietary interventions focused on reducing the dietary inflammatory index may configure a novel approach for the prevention of glycemic disorders.

## Association of prediabetes with complications

While the association of diabetes with microvascular and macrovascular complications is well known, less well known is the relationship of these complications with prediabetes. However, a growing body of evidence suggests that prediabetes is associated with an increased risk of both microvascular and macrovascular complications (4). Wang et al. in this special issue explored the association between estimated glomerular filtration rate (eGFR) and incident IFG in the Chinese population with normal FPG at baseline. They showed an association between reduced renal function and increased glomerular perfusion with an increased risk of developing IFG. This study illustrates that glomerular hyperfiltration may be a common manifestation of kidney dysfunction with recent progression from normoglycemia to prediabetes.

Wang et al., evaluated the association between serum levels of Klotho (a membrane protein originally described to possess anti-aging properties) and the prevalence of diabetes among adults in the United States. They found a nonlinear and positive association between serum Klotho levels and the prevalence of diabetes. Given the potential role of the Klotho and FGF23 signaling pathways on cardiovascular and renal function (5, 6), further studies evaluating the role of Klotho across the continuum of glycemic dysfunction may offer new approaches to prevent cardio-renal complications in both prediabetes and diabetes.

One of the most overlooked complications of hyperglycemia is periodontitis (7). Wang et al. used a Mendelian randomization approach to investigate the causal relationship between fasting glucose and periodontitis. Their findings confirm previous observations (7) and provide additional evidence to support a causal effect of hyperglycemia on periodontitis. This study underscores the importance of prevention, early detection, and timely treatment of periodontitis in patients with prediabetes and diabetes.

## New therapeutic pathways for treatment of prediabetes

Cloro et al. assessed the effects of sacubitril/valsartan, a combined angiotensin receptor blocker and inhibitor of neprilysin (an enzyme that degrades several vasoactive peptides, including natriuretic peptides), on glycemic and metabolic parameters, insulin resistance, and echocardiographic parameters in patients with prediabetes and heart failure with reduced ejection fraction (HFrEF). Their results suggest that sacubitril/valsartan improves metabolic control and insulin resistance in this population. These results are consistent with a meta-analysis that demonstrated the efficacy of angiotensin-converting enzyme inhibitors or angiotensin II receptor blockers in reducing the risk of new-onset type 2 diabetes (8). The increase in natriuretic peptides with neprilysin inhibition may further promote insulin sensitivity and improve glycemic control (9), as elegantly discussed in the paper by Cloro et al. Further studies should evaluate if neprilysin inhibition could have a role in preventing or treating prediabetes in individuals without heart failure.

## Future perspectives and unmet needs

Prediabetes research has much to uncover (**Figure 1**). Future studies should focus on: 1) creating effective strategies to increase screening and identification of prediabetes by primary care providers; 2) increasing availability of and referral to diabetes prevention programs (10); 3) introducing diagnostic methods that decrease testing burden and improve prediction of progression to type 2 diabetes, complications and mortality (e.g., 1-hour OGTT) (11); 4) refining stratification of the risk of cardio-renal complications and likelihood of progression to diabetes; and 5) understanding the role of emerging drugs in preventing cardiovascular complications and diabetes development (e.g., SGLT2 inhibitors, GLP-1 receptor agonists, and GLP-1/GIP- dual agonists). We encourage researchers to prioritize studies on prediabetes, as interventions in this phase may yield greater long-term health benefits compared to those with more advanced stages of glycemic dysfunction.

## Author contributions

All authors listed have made a substantial, direct, and intellectual contribution to the work and approved it for publication.
